# Results From the Third and Fourth WHO External Quality Assessments for the Molecular Detection of Respiratory Syncytial Virus

**DOI:** 10.1111/irv.70302

**Published:** 2026-08-02

**Authors:** Fernando do Couto Motta, Obadiah Kenji, Ian Barr, Shabana Bi, Anne von Gottberg, Jean‐Michel Heraud, Siddhi Hirve, Sanjiv Rughooputh, Nicole Wolter, Maria Zambon, Thomas C. Williams, Wenqing Zhang

**Affiliations:** ^1^ Oswaldo Cruz Institute, FIOCRUZ Rio de Janeiro Brazil; ^2^ Global Influenza Programme World Health Organization Geneva Switzerland; ^3^ WHO Collaborating Centre for Reference and Research on Influenza, VIDRL Peter Doherty Institute for Infection and Immunity Melbourne Australia; ^4^ Department of Microbiology and Immunology University of Melbourne Melbourne Australia; ^5^ UKHSA London UK; ^6^ UK NEQAS for Microbiology London UK; ^7^ National Institute for Communicable Diseases of the National Health Laboratory Service Johannesburg South Africa; ^8^ School of Pathology, Faculty of Health Sciences University of the Witwatersrand Johannesburg South Africa; ^9^ University of Edinburgh Edinburgh UK

**Keywords:** disease control, EQA, laboratory capacity, laboratory diagnostics and systems, quality, respiratory diseases, RSV

## Abstract

**Background:**

In 2016, the World Health Organization (WHO) initiated the global RSV surveillance programme, which aimed to strengthen laboratory diagnostic capacity and evaluate molecular detection test performance. External quality assessments (EQAs) are conducted regularly as part of this programme to ensure ongoing laboratory proficiency. The third (2021–2022) and the fourth (2023–2024) WHO RSV EQAs were conducted to evaluate the ability of participating laboratories to accurately detect and subtype RSV.

**Methods:**

For each EQA panel, 12 lyophilised RSV isolates were prepared using both recently circulating and RSV strains used in previous EQAs. The isolates were shipped to 81 and 85 laboratories in the third and fourth EQA, respectively. Participants tested the specimens using their routine molecular diagnostic protocols and reported the results through the UK NEQAS online platform. Performance was evaluated based on detection and subtyping accuracy, and a scoring system was used that ranged from *good performance* (24/24 correct results) to *unacceptable* (≤ 16 correct results).

**Results:**

A total of 73 (90.1%) and 74 (87.1%) laboratories returned results for the third and fourth RSV molecular EQAs, respectively. In the third EQA, 69.9% of laboratories were classified as good, 15.1% as acceptable and 1.4% as satisfactory, resulting in 86.3% within the desirable range; 12.3% were unacceptable and 1.4% poor. In the fourth EQA, 60.8% were good, 28.4% acceptable and 2.7% satisfactory, with 91.9% in the desirable range; 8.1% were unacceptable, and none were poor.

**Conclusion:**

The third and fourth WHO RSV EQA demonstrated good overall laboratory performance for RSV molecular detection and subtyping. These findings highlight the importance of EQA to document high‐quality diagnostics, especially with the introduction of RSV vaccines and monoclonal antibodies. Ongoing EQAs, including the first WHO RSV sequencing EQA initiated in 2024, will further enhance global laboratory capacity for RSV surveillance and outbreak response.

## 1 Introduction

Respiratory syncytial virus (RSV) is a nonsegmented RNA virus responsible for seasonal respiratory epidemics, commonly associated with the autumn‐winter seasons in temperate regions. Most infections present with mild respiratory symptoms and can be managed without hospitalisation; however, a proportion of cases present with more severe symptoms leading to hospitalisation. Globally, RSV is estimated to cause 33.0 million acute lower respiratory tract infections annually in children under 5 years old, resulting in 3.6 million hospitalisations and 101,400 RSV attributable deaths, the vast majority of which are in low‐ and middle‐income countries [1]. A scarcity of data from limited resources countries, together with the imminent arrival of new preventative measures for RSV (maternal immunisation and long‐acting monoclonal antibodies), led in 2016 to the establishment of the World Health Organization (WHO) Global RSV surveillance programme [2]. Participating laboratories were selected from those participating in the Global Influenza Surveillance and Response System (GISRS) [3] at that time. One of the aims of this pilot was to evaluate existing and new RSV molecular tests in all six WHO Regions. To achieve this, in 2016, the first external quality assessment (EQA) exercise for the molecular detection of RSV was successfully developed, launched and completed [4].

Recognising that a more detailed characterisation of RSV molecular epidemiology was likely to be required with the roll‐out of new interventions, in 2019, the WHO, in collaboration with the RSV Reference Laboratories, Public Health England, and UK NEQAS, undertook the second EQA exercise. The primary objective of the second EQA in 2019 was to ensure that participating laboratories could detect and subtype recently circulating RSV viruses. This was a change to the first WHO RSV EQA (2016–2017), which assessed the proficiency of the molecular detection of RSV only and not subtyping [4]. The composition of the RSV EQA 2019 panel was based on selected representative viruses from different regions of the world, and 28 national laboratories involved in Phase 2 of the RSV Surveillance Program successfully completed it.

The COVID‐19 pandemic, which began in Wuhan, China, at the end of 2019 [5], was associated with disruption to the work of diagnostic laboratories globally [6]. It was also associated with a temporary decrease in RSV infection rates, likely due to the lockdown measures and nonpharmaceutical interventions implemented to prevent the transmission of SARS‐CoV‐2 [7]. However, as RSV infections rebounded following the easing of lockdown measures and the value of robust molecular diagnostics became evident in the wake of the pandemic, a third and fourth round of EQA was planned for 2021–2022 and 2023–2024, respectively, with a substantial increase in the number of participating laboratories to 73 and 74 in the third and fourth EQA, respectively, including the involvement of the European Centre for Disease Prevention and Control (ECDC) national laboratories. Here, we describe the results of the third and fourth EQAs, which aimed to evaluate national laboratories' capability to detect and subtype RSV, outline the testing process and assess the timeliness of responses.

## 2 Methods

### 2.1 Composition of the Third and Fourth RSV EQA Panels

The third RSV EQA conducted in 2021–2022 comprised 12 samples and was developed by considering the recently circulating RSV viruses as well as the isolates used in the second RSV EQA (Table 1). The RSV Reference Laboratories evaluated whether the panel composition should be updated to reflect more recent RSV viruses that had emerged since the previous EQA. It was agreed that incorporating new viruses would not significantly affect the performance of the existing primers and probes. However, several changes were made to the third edition panel. The RSV‐A hRSV/A/Australia/VIC‐RCH010/2018 and the RSV‐B hRSV/B/South_Africa/NICD‐R06224/2019 isolates were removed. In their place, isolates of hRSV/A/England/174460397/2017 and hRSV/B/Australia/VIC‐VIDRL003/2015 were used directly (Sample Numbers 7125 and 7126, respectively) and diluted by 10^−2^, to create higher cycle threshold (*Ct*) replicates (Sample Numbers 7125 and 7128 respectively) comparable to the original samples. Finally, one of the two negative controls (Sample Number 7124) was produced by mixing an influenza A(H1N1)pdm09 isolate from 2015 (A/Scotland/P2/2015), provided by the Crick Worldwide Influenza Centre, which is a WHO Collaborating Centre for Reference and Research on Influenza, with a SARS‐CoV‐2 isolate from the Gamma lineage (hCov/Porton/B1.1.28.1, supplied by UKHSA). The second control was a freeze‐dried matrix, negative for both RSV‐A and RSV‐B (Sample Number 7129). This revised panel provided a robust and comprehensive evaluation of RSV diagnostic performance, taking into account the broader context of the pandemic response.

**TABLE 1:** Viral isolates included in the third RSV EQA.

Sample ID	Isolate name (standard nomenclature) [8]	Subtype	GISAID ID	Expected *Ct* value
7124	SARS‐CoV‐2 (Gamma lineage) + Influenza A(H1N1)pdm09	N/A	N/A	N/A
7125	hRSV/A/England/174460397/2017	A	EPI_ISL_732338	24.9^a^
7126	hRSV/B/Australia/VIC‐VIDRL003/2015	B	EPI_ISL_4569432	26.9
7127	hRSV/B/South Africa/NICD‐R05898/2019	B	EPI_ISL_9003920	25.0
7128	hRSV/B/Australia/VIC‐VIDRL003/2015	B	EPI_ISL_4569432	33.0^a^
7129	Freeze‐dried matrix, RSV‐A and RSV‐B negative	N/A	N/A	N/A
7130	hRSV/A/Australia/VIC‐VIDRL002/2018	A	EPI_ISL_4602779	21.3
7131	hRSV/A/Australia/VIC‐RCH010/2018	A	EPI_ISL_1834085	23.1
7132	hRSV/B/South Africa/NICD‐R06224/2019	B	EPI_ISL_9003920	25.9
7133	hRSV/A/South Africa/NICD‐R06229/2019	A	EPI_ISL_9003918	22.5
7134	hRSV/B/England/180440410/2018	B	EPI_ISL_732354	23.1
7135	hRSV/A/England/174460397/2017	A	EPI_ISL_732338	17.8

*Note:* Ct values were generated by UK NEQAS using the Altona RealStar RSV PCR assay.

Abbreviation: N/A, not applicable.

^a^ 10^−2^ dilution of original sample.

The fourth RSV EQA conducted in 2023–2024 was designed to maintain continuity with previous panels while incorporating isolates that reflect recent RSV diversity. The panel comprised 12 samples, including two negative controls (one water control and one containing SARS‐CoV‐2 Omicron and seasonal influenza A), eight RSV‐positive samples (four RSV‐A and four RSV‐B) and two diluted samples to test assay sensitivity at lower viral concentrations (Table 2). To ensure representation across regions and continuity with earlier panels, one RSV‐A (hRSV/A/South Africa/NICD‐R06229/2019) and one RSV‐B (hRSV/B/England/180440410/2018) isolate were carried over from the previous EQA. The remaining isolates were newly selected from recent collections in Australia and South Africa, chosen based on genomic diversity using p‐distance analysis and lineage classification via NextClade. This panel provided a balanced mix of reference and contemporary isolates, ensuring robust evaluation of RSV molecular diagnostics across both established and emerging viral lineages.

**TABLE 2:** Viral isolates included in the fourth RSV EQA.

Sample ID	Isolate name (standard nomenclature)	Subtype	GISAID ID	Expected *Ct* value
2512	hRSV/A/Australia/VIC‐MMC055/2021	A	EPI_ISL_2543777	22.0
2513	hRSV/B/Australia/NT‐RDH0763/2022	B	EPI_ISL_16714812	27.0
2514	Negative control	N/A	N/A	N/A
2515	hRSV/B/Australia/NT‐RDH0085/2023	B	EPI_ISL_18971823	28.0
2516	hRSV/B/Australia/NT‐RDH0085/2023	B	EPI_ISL_18971823	28.0
2517	hRSV/A/Australia/NT‐RDH0109/2023	A	EPI_ISL_18972143	25.0
2518	hRSV/B/Australia/NT‐RDH0426/2022	B	EPI_ISL_15896258	23.0
2519	hRSV/A/South Africa/NICD‐R06229/2019	A	EPI_ISL_9003918	32.0
2520	hRSV/A/Australia/VIC‐MMC045/2020	A	EPI_ISL_2543773	26.0
2521	H1N1A/England/73/2022 and Omicron BA.4‐19/114	N/A	N/A	N/A
2522	hRSV/A/Australia/VIC‐MMC045/2020	A	EPI_ISL_2543773	20.0
2523	hRSV/B/England/180440410/2018	B	EPI_ISL_732354	29.5

### 2.2 Analysis of Possible Primer/Probe Mismatches

To determine whether the EQA isolates contained genetic mutations that could affect the performance of the recommended primers and probes for RSV detection, we conducted an in silico analysis of three primer sets endorsed by the national laboratories participating in the EQA. These included the CDC Pan RT‐PCR targeting the Matrix (M) gene [9], the VIDRL Multiplex RT‐PCR targeting the Polymerase (L) gene [10] and the CDC Duplex RT‐PCR targeting the Nucleocapsid (N) gene [11]. This analysis, which was initially performed on the RSV isolates used in the second (2019–2020) EQA, was also applied to the new isolates provided by the reference laboratories for the third round. This analysis was not conducted for the fourth EQA, as most of the isolates included in the third EQA were also incorporated into the fourth EQA.

### 2.3 Preparation of the Panel

Viruses included in the EQA panels were propagated in Hep‐2 cells (a HeLa derivative, European Collection of Authenticated Cell Cultures 86030501) in growth media (Eagle Minimum Essential Medium‐Ref Sigma M4655‐containing Earle's salt, L‐glutamine and sodium bicarbonate supplemented with 10% foetal bovine serum, Lonza Bioscience Ref. DE 14‐801F), harvested and then used to prepare the EQA isolates that were subsequently lyophilised. On successful completion of predistribution testing and quality control checks, the specimens were labelled and packed for dispatch by UK NEQAS. The panels were stored and shipped at room temperature.

### 2.4 Reconstitution of the Panel

Laboratories were advised to store the lyophilised specimens preferably at 4°C until reconstitution. Lyophilisates were reconstituted in 1.2 mL of sterile molecular grade water. For nucleic acid extraction, participants were instructed to use their existing laboratory assays and kit instructions. Once reconstituted, samples were to be extracted, PCR performed and residual RNA stored at −80°C.

### 2.5 Molecular Detection and Subtyping of RSV‐A and RSV‐B Using Real‐Time RT‐PCR

The Pan RSV assay [9], RSV rRT‐PCR duplex assay from CDC [11] and the multiplex subtyping assay from VIDRL [10] were the three WHO RSV Program recommended protocols for RSV detection and subtyping. Nevertheless, participating laboratories could decide to use any commercial or in‐house assay to perform the EQA testing. The protocol used was requested to be recorded on the general information form to be submitted along with the test results.

### 2.6 Reporting of Results

Laboratories reported their EQA results through the UK NEQAS online platform under individualised logins and passwords. Besides the results and *Ct* values for each sample in the panel, the participant laboratories agreed to answer a standard survey designed by UK NEQAS and the RSV Reference Laboratories. This survey collected information on general laboratory practices, nucleic acid extraction, RSV molecular detection assay and amplification platforms used to perform the EQA.

### 2.7 Scoring and Analysis of Results

The same scoring system as used in the second EQA (2019) was used in the third and fourth EQAs (Table 3). Results for each laboratory, for each sample, were classified into the following categories: RSV (not subtyped), RSV (correctly subtyped), RSV (incorrectly subtyped) and others, including no virus, indeterminate or additional pathogens.

**TABLE 3:** Scoring scheme for the third and fourth WHO RSV EQA.

Description	Report text	Score
RSV identified	RSV	1
RSV‐A/B identified	RSV‐A/B	1
RSV negative	RSV not detected	1
RSV negative	RSV‐A/B subtypes not detected	1
No virus	RSV detected	0
RSV wrong subtype	RSV wrong subtype	0
Indeterminate	Indeterminate	0
Named virus other than that specified	Named virus	0
RSV + an additional pathogen	Additional pathogen	0

The interpretation of the scores was as follows: Each EQA panel included 12 samples, and results were scored at two levels—RSV detection (detected or not detected) and RSV subtyping (RSV‐A or RSV‐B, if applicable)—with one point awarded for each correct detection and one point for each correct subtype, yielding a maximum total of 24 points. A score of 24/24 was interpreted as a good performance, scores between 22–23 as acceptable; 20–21 as satisfactory; 17–19 as poor; and 16 or less as unacceptable. Individual laboratory reports containing all analyses of submitted results (score, performance rate, time to reporting and general laboratory information) compared to the overall laboratory scores were made available to participating laboratories through the NEQAS platform.

The overall performance score for detection was calculated by summing the number of correct RSV identifications submitted by all participating laboratories for each individual sample and then expressing this as a percentage of the total number of possible results (i.e., the number of laboratories that submitted results for each EQA multiplied by the number of specimens; 73 × 12 = 876 for the third EQA and 74 × 12 = 888 for the fourth EQA). Similarly, for subtyping, the overall performance score was determined by adding the number of correct RSV‐A and RSV‐B subtype identifications provided by all laboratories for each sample and then expressing this as a percentage of the total possible results. To calculate the combined overall score for both detection and subtyping, the number of correct results obtained across all laboratories and samples was summed and expressed as a percentage of the total possible results.

## 3 Results

### 3.1 Details of Laboratories Participating in the Third and Fourth EQA and Time Taken to Report Results

Eighty‐one laboratories in 70 countries from all six WHO regions were invited to participate in the third EQA. Of these, 73 (90.1%) reported results (Figure S1). The median time the laboratories took to report the results was 64 days (range 6–191 days). For the fourth EQA, 85 laboratories from 69 countries were invited, and 74 (87.1%) laboratories returned results (Figure S1B). The median time to report the results for this fourth EQA was 94 days (range 18–137 days). For most laboratories that did not report results, this was due to specimens being delayed in customs clearance or because logistical arrangements and paperwork were incomplete, preventing dispatch.

**FIGURE 1:**
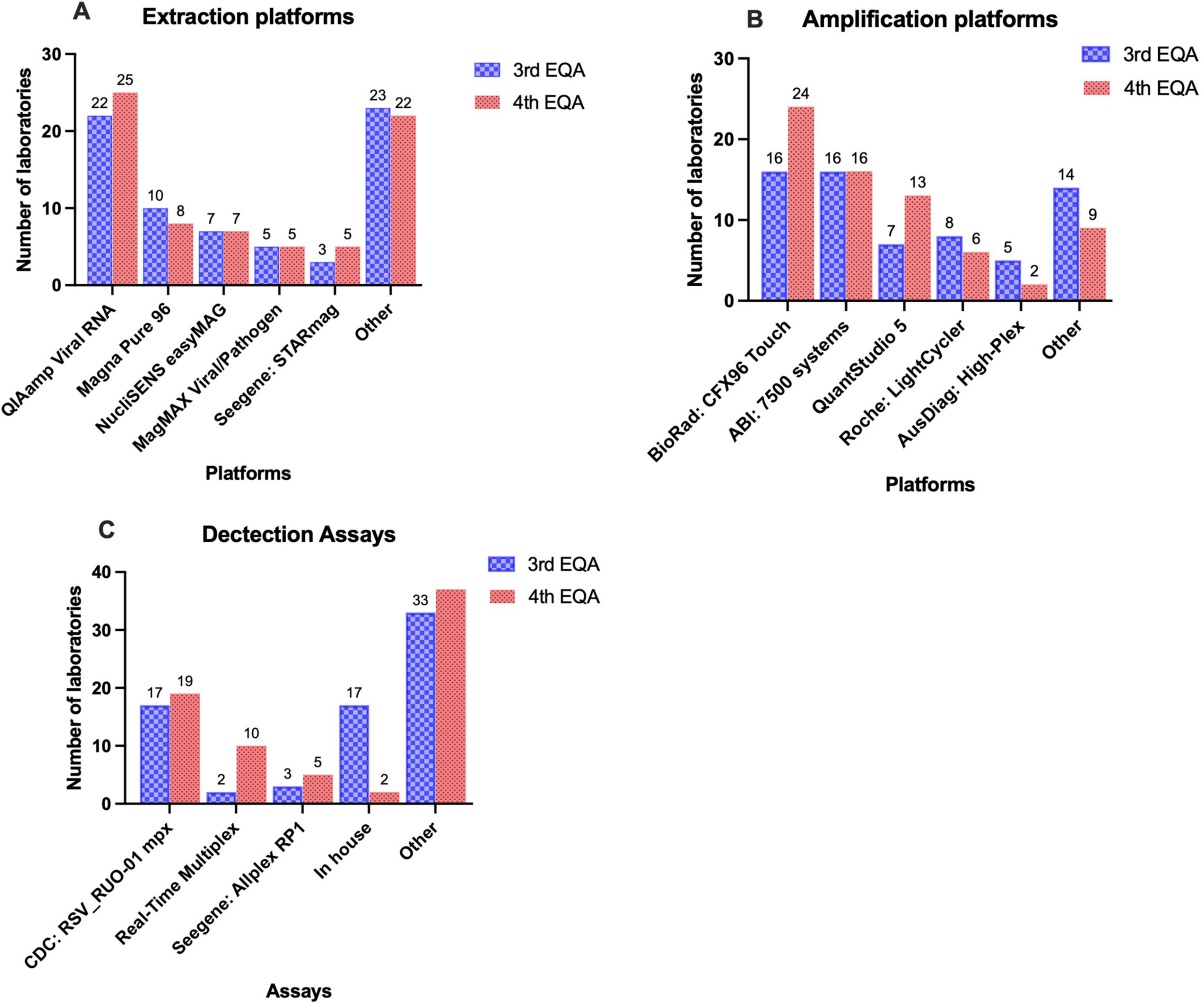
Distribution of extraction platforms (A), amplification platforms (B) and detection assays (C) reported by laboratories participating in the third and fourth WHO RSV molecular EQAs. The *y* axis represents the number of laboratories reporting each platform or assay. Figure 1A shows the distribution of nucleic acid extraction methods, including the QIAamp Viral RNA kit (Qiagen), Magna Pure 96 (Roche), NucliSENS easyMAG (bioMérieux), MagMAX Viral/Pathogen (Thermo Fisher Scientific), Seegene STARmag (Seegene) and other commercial or in‐house methods. Figure 1B shows the distribution of real‐time PCR amplification platforms, including the CFX96 Touch Real‐Time PCR Detection System (Bio‐Rad), ABI 7500 Real‐Time PCR Systems (Applied Biosystems), QuantStudio 5 (Thermo Fisher Scientific), LightCycler (Roche), and other instruments. Figure 1C shows the distribution of RSV detection assays, including the CDC pan‐RSV rRT‐PCR assay (Centers for Disease Control and Prevention; RUO‐01 multiplex), Real‐Time multiplex assays, Allplex Respiratory Panel 1 (Seegene), in‐house and other assays. *Note:* The total number of reporting laboratories differs across extraction platforms, amplification platforms and detection assays because some laboratories did not complete the survey for all the categories.

The Qiagen QIAamp Viral RNA kit was the most frequently utilised method for nucleic acid extraction, accounting for 31.4% (22/70) and 33.7% (25/72) of responses in the third and fourth EQA, respectively (Figure 1). The most commonly used amplification platforms in the third EQA were the Bio‐Rad CFX 96 Touch and ABI 7500 from Thermo Fisher Scientific, each reported by 16/66 users (24.3%). The Bio‐Rad CFX96 was still prominent in the fourth EQA (34.2%, 24/70), whereas the ABI 7500 was used by 22.8% (16/70) of the laboratories. A total of 24 and 25 different detection assays were used in the third and fourth EQA, respectively. The CDC pan‐RSV assay and in‐house assay were the most frequently reported in the third EQA (23.6%, 17/72 each). The CDC pan‐RSV assay was also the most commonly reported in the fourth EQA (26%, 19/73).

### 3.2 Performance Rating and Scores

**FIGURE 2:**
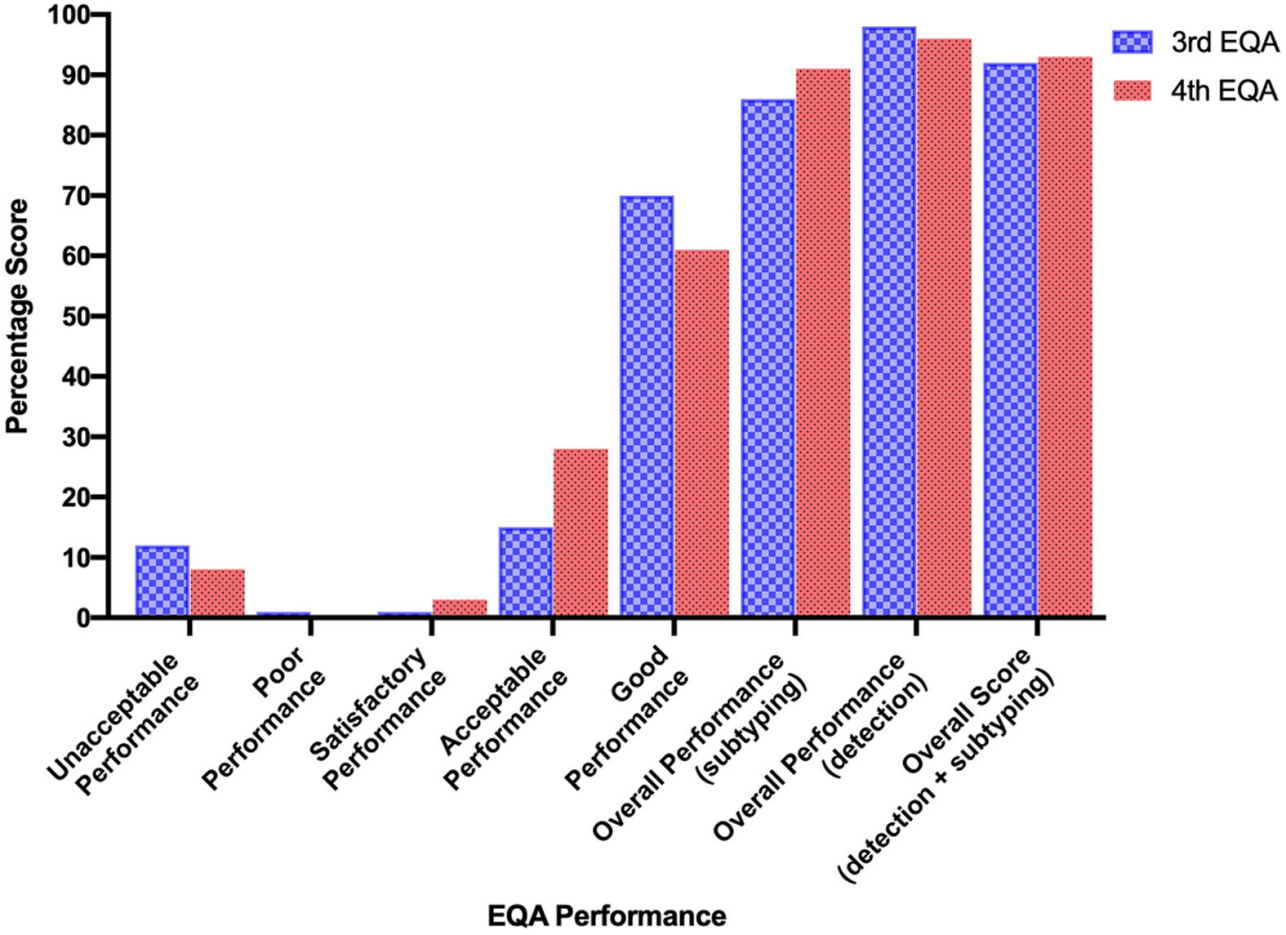
Comparison of performance by participating laboratories in the third and fourth WHO RSV Molecular External Quality Assessments. This figure shows the distribution of laboratory performance ratings across the two EQA rounds, categorised into five performance tiers: good, acceptable, satisfactory, poor and unacceptable. Scores were derived from the correct detection and subtyping of 12 samples for each EQA round, and performance classifications were based on established scoring thresholds. The figure also illustrates the overall performance score categories, with separate bars for the third and fourth EQA. The overall performance score represents the combined accuracy of RSV detection and subtype assignment across all challenge samples and is presented as a summary measure to facilitate comparison of laboratory performance between EQA rounds. The *Y* axis represents the percentage score.

Out of 73 laboratories returning results in the third EQA, 51 laboratories (69.9%) were classified as having a good performance, 11 (15.1%) were classified as acceptable and 1 (1.4%) as satisfactory, totalling 63 (86.3%) laboratories within the desirable range (Figure 2). A total of 10 (13.7%) laboratories performed below the satisfactory threshold (score < 20), with one performing poorly and 9 (12.3%) receiving ‘unacceptable’ scores (≤ 16).

Out of 74 laboratories returning results in the fourth EQA, 45 laboratories (60.8%) were classified as having a good performance, 21 (28.4%) were classified as acceptable and 2 (2.7%) as satisfactory, totalling 68 (91.9%) laboratories within the desirable range. A total of 6 (8.1%) laboratories performed below the satisfactory threshold (score < 20), with all receiving ‘unacceptable’ scores (≤ 16) and none with ‘poor’ score.

Regarding overall detection and subtyping performance, the third EQA reported an RSV detection score of 98.0% (858/876) and an RSV subtyping score of 86.3% (756/876). The overall score of both molecular detection and subtyping combined was 92.1% (1614/1752). Meanwhile, in the fourth EQA, the overall score for RSV detection was 96.3% (855/888) and 90.7% (805/888) for RSV subtyping. The overall score of both molecular detection and subtyping combined was 93.5% (1660/1776).

### 3.3 Error Patterns and Sample‐Specific Results

**FIGURE 3:**
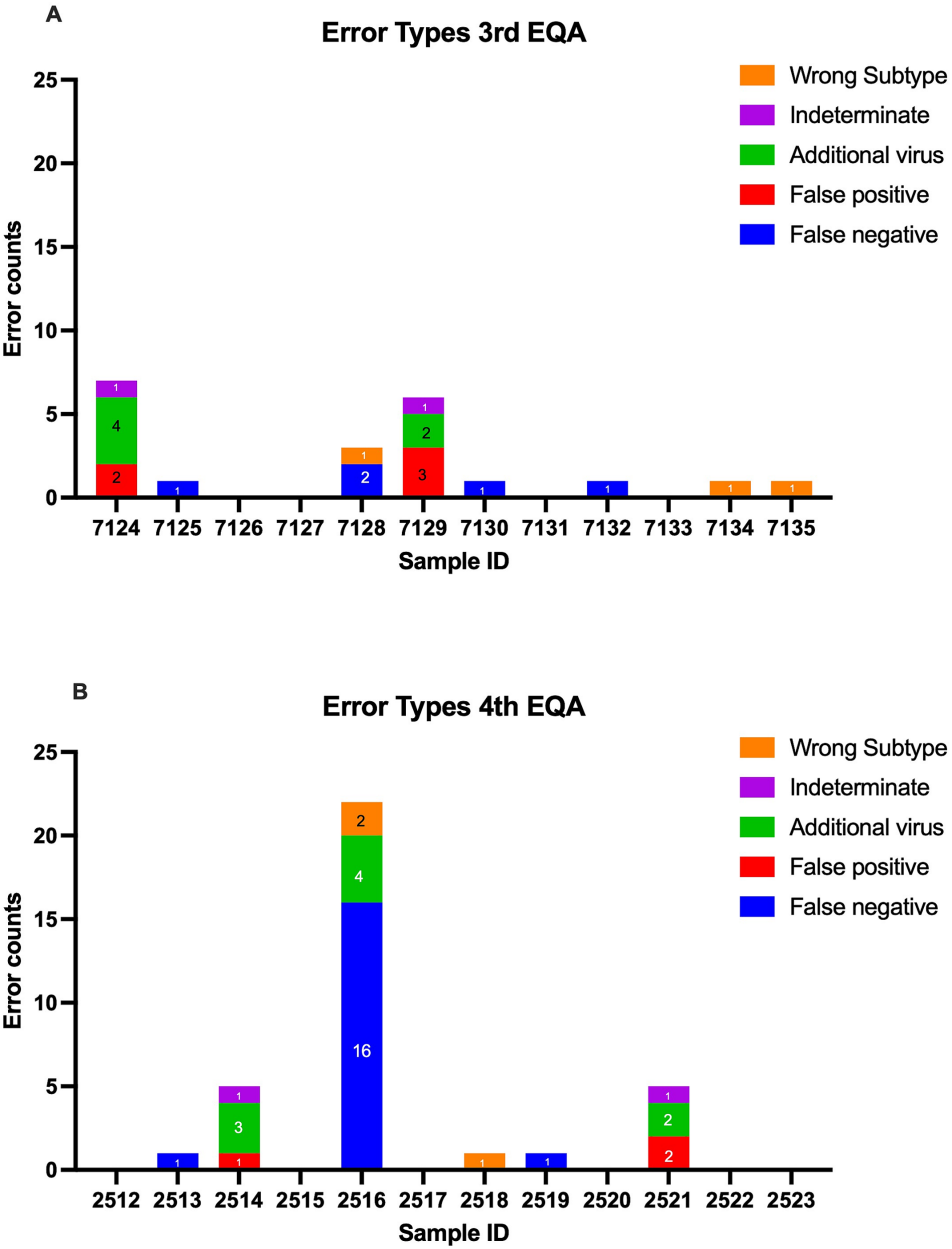
Error types for the third (A) and fourth (B) External Quality Assessments. This figure presents the distribution of error types observed for each sample across the third (Panel A) and fourth (Panel B) RSV EQAs. Error categories include false negatives, false positives, additional virus detection, indeterminate results and wrong subtype assignment. Each bar represents the total number of errors reported for a given sample, with colours distinguishing error types.

In the third EQA, most of the participating laboratories accurately detected and subtyped RSV samples (Figure 3A). However, Sample 7124, the mixed‐virus negative control (Influenza A and SARS‐CoV‐2 positive), was the most error‐prone, resulting in two false RSV positives, four instances of additional pathogen reporting (not the relevant pathogen included) and one indeterminate result. Sample 7129, a true negative sample, produced three false RSV positives, two reports of additional viruses and one indeterminate result. Samples 7125, 7130 and 7132 had one false negative result each, and Samples 7134 and 7135 had one incorrect subtyping result each. Sample 7128 had the highest number of false negative results (2) and one wrong subtyping result. This sample had the highest mean reported *Ct* value (31.9). All other samples in this round were correctly identified by all participants, and no consistent link was found between the mean reported *Ct* value and detection accuracy (Table S1).

In the fourth EQA, the most challenging sample was 2516 (RSV‐B positive) with 16 false negatives, 2 subtype errors and 4 reports of additional viruses, making it the highest error sample across both EQAs (Figure 3B). This sample had a predistribution *Ct* value of 28.4. However, the mean *Ct* value for laboratories that reported results was 35.8 (range 29.6–40.0). Sample 2521, the mixed‐virus negative control (Influenza A and SARS‐CoV‐2 positive), had two false positives, two additional virus reports and one indeterminate result. Sample 2514, the true negative sample, produced one false positive, three additional virus reports and one indeterminate. Sample 2518 had one wrong subtype report, whereas Samples 2513 and 2519 had one false negative result each. The remaining samples (2512, 2515, 2517, 2520, 2522 and 2523) were consistently reported correctly with no errors.

### 3.4 *Ct* Value Distribution and Detection Scores

The mean *Ct* values reported by participating laboratories were generally consistent with the reference laboratory *Ct* values (Table S1). For samples with reference laboratory *Ct* values below 30, most laboratories reported mean *Ct* values within one to three cycles of the reference value. Figure S2 illustrates the distribution of *Ct* values reported by participating laboratories across both EQAs. The figure highlights the tight clustering of Ct values for most RSV‐positive samples.

## 4 Discussion

Many laboratories participated in the WHO RSV EQA, with 73 (90.1%) and 74 (87.1%) of laboratories returning their results in the third and fourth EQA, respectively. Overall performance was good, with 98% of RSV‐containing samples detected and 86% of RSV samples correctly subtyped in the third EQA. In the fourth EQA, the overall score for RSV detection was 96% and 91% for RSV subtyping. In the third EQA, 86% of laboratories achieved scores within the desirable range, increasing to 92% in the fourth EQA.

These findings are consistent with those reported during the first and second WHO RSV EQAs. The first EQA (2016–2017), conducted during the pilot phase of the WHO Global RSV Surveillance Programme, demonstrated that participating laboratories could successfully implement standardised molecular detection methods, with 84% of laboratories correctly identifying all panel specimens [4]. The second EQA (2019–2020), which expanded participation from 14 to 28 laboratories and introduced contemporary RSV strains together with RSV subtyping, reported detection and subtyping accuracies of 98.8% and 99.6%, respectively, confirming the ability of laboratories to accurately detect both historical and recently circulating RSV strains [12]. The present study demonstrates that these high standards have been maintained despite a substantial increase in the number of laboratories participating and continued implementation of different commercial and laboratory‐developed molecular assays.

The sample with the highest *Ct* value (lowest viral load) had a higher number of false negatives reported, whereas false positives were reported for the SARS‐CoV‐2/Influenza A and matrix negative controls. In the third EQA, Sample 7128 (reference lab *Ct* 33.0 and mean reported *Ct* 31.92) accounted for most errors, whereas Sample 2516 in the fourth EQA (reference lab *Ct* 28.4 and mean reported *Ct* 35.8) was the most error‐prone overall, with 16 false negatives and multiple subtype and additional pathogen reporting errors. This suggests that viral concentration remains a crucial factor influencing diagnostic sensitivity. To minimise false negatives in routine practice, laboratories should implement stringent quality control measures—such as monitoring extraction efficiency and ensuring proper specimen transport and storage, particularly for field samples where RNA degradation may occur due to suboptimal conditions. Incorporating appropriate internal controls can help flag low quality samples.

Conversely, false positive RSV results and additional pathogen detection, on the other hand, tended to arise from negative control samples, indicating a need for ongoing vigilance against nonspecific amplification and cross‐reactivity or contamination. The use of no‐template controls and proper separation of preamplification and postamplification areas are essential to minimise such risks. These findings reinforce the importance of maintaining robust contamination prevention strategies across all PCR‐based testing, not only for RSV diagnostics, as molecular testing has become a core laboratory capability. They also highlight the value of external quality assessment programmes in providing independent assessment, identifying opportunities for corrective action and fostering a culture of continuous quality improvement in laboratories.

The reported *Ct* values were generally consistent with the reference laboratory *Ct* values, especially for samples with low Ct values. Variability in *Ct* values was observed in low‐positive samples such as 7128, 2513 and particularly 2516, aligning with observed detection errors. This consistency supports the overall robustness of assay performance, while reinforcing the need for careful validation of protocols against low‐concentration specimens.

An important feature of the third and fourth EQAs is the continued inclusion of contemporary RSV strains representative of viruses circulating globally. RSV continues to evolve through the accumulation of mutations, including within genomic regions targeted by molecular diagnostic assays, underscoring the importance of ongoing genomic surveillance to ensure continued assay performance [13, 14]. Genetic changes may introduce primer or probe mismatches that reduce assay sensitivity if molecular assays are not periodically evaluated and updated [12, 14]. The continued high EQA performance observed in the current study suggests that widely used commercial and in‐house assays remain suitable for detecting the contemporary RSV strains included in the EQA panel, supporting previous findings that regular updating of EQA panels with genetically representative circulating viruses is an effective strategy for maintaining diagnostic quality [12].

A significant proportion of laboratories that participated in both EQAs used commercial kits for RSV detection and subtyping. However, little information about the genetic targets for these primers and probes is publicly available. Therefore, it is difficult to evaluate whether these primers/probes had mismatches against the viruses included in the EQA; however, overall good molecular detection performance regardless of approach used suggests no issue with any of the widely used commercial kits.

This exercise highlights the importance of EQAs to maintain high laboratory standards for correctly detecting RSV and other respiratory viruses and for comparing individual laboratories' results with others to ensure they are obtaining accurate results for their rRT‐PCR testing. The fourth RSV EQA for the detection and subtyping of RSV was distributed in the second quarter of 2024 and included an EQA for RSV whole genome sequencing (WGS).

## 5 Conclusion

The third and fourth WHO EQAs for molecular detection and subtyping of RSV showed high overall performance, with the majority of participating laboratories accurately detecting and subtyping RSV. Nevertheless, there remained variability in the ability to detect RSV in samples with high *Ct* values. Building on these experiences, planning for a fifth molecular detection/subtyping and second WGS EQA panels is under consideration. Accurate RSV diagnostic testing is likely to become increasingly important to assess burden of disease, impact of vaccination and RSV interventions. Additionally, accurate RSV testing is likely to be needed to detect breakthrough infections in the infants of pregnant women receiving RSV vaccines, and newborns receiving nirsevimab, to ensure the individual and public health expectations are maintained.

## Author Contributions

**Fernando do Couto Motta:** conceptualization, writing – original draft, writing – review and editing, formal analysis, methodology, investigation, visualization. **Obadiah Kenji:** conceptualization, investigation, writing – original draft, methodology, writing – review and editing, project administration, formal analysis, visualization. **Ian Barr:** writing – review and editing, methodology. **Shabana Bi:** writing – review and editing, formal analysis, methodology. **Anne von Gottberg:** writing – review and editing, methodology. **Jean‐Michel Heraud:** writing – review and editing, methodology. **Siddhi Hirve:** writing – review and editing, supervision, funding acquisition, resources. **Sanjiv Rughooputh:** writing – review and editing, supervision, formal analysis, conceptualization, project administration, methodology. **Nicole Wolter:** writing – review and editing, methodology. **Maria Zambon:** writing – review and editing, methodology. **Thomas C. Williams:** conceptualization, writing – review and editing, methodology, formal analysis, writing – original draft. **Wenqing Zhang:** supervision, resources, funding acquisition, writing – review and editing.

## Funding

This work was supported by the Bill and Melinda Gates Foundation (78084).

## Conflicts of Interest

The authors declare no conflicts of interest.

## Supporting information


**Figure S1A:** Countries submitting results for the third (2023) EQA.

**Figure S1B:** Countries submitting results for the fourth (2024) EQA.

**Figure S2:** Ct value distribution of all participants in the third and fourth EQAs.

**Table S1:** Sample intended results, Ct values, and detection scores for the third and fourth EQAs.

## Data Availability

The data that support the findings of this study are available on request from the corresponding author. The data are not publicly available due to privacy or ethical restrictions.
