# Supplementary figures and images for: Results From the Third and Fourth WHO External Quality Assessments for the Molecular Detection of Respiratory Syncytial Virus
Source: Influenza Other Respir Viruses. 2026 Aug 2;20(8):e70302. doi: 10.1111/irv.70302 (PMC13429278; doi:10.1111/irv.70302)

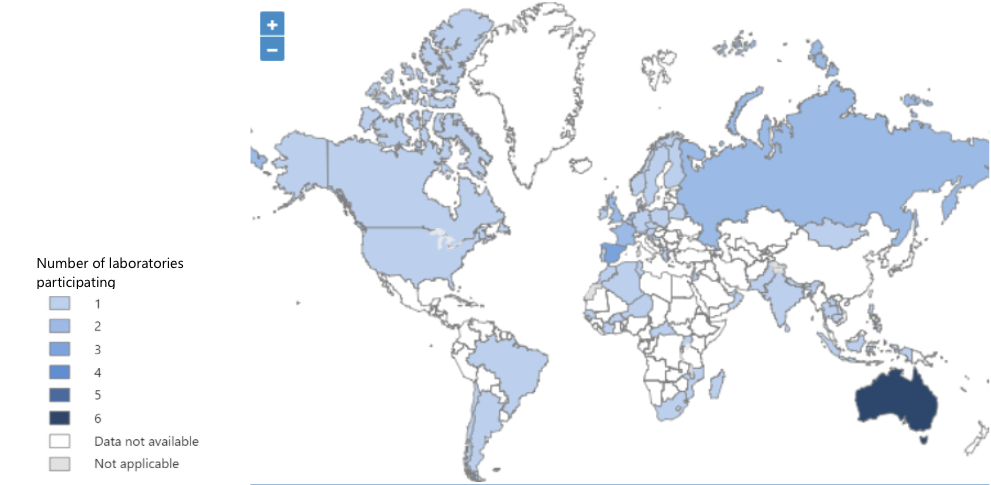

Supplement: Supplementary file 1 — Figure S1A: Countries submitting results for the third (2023) EQA. [file IRV-20-e70302-s004.png]

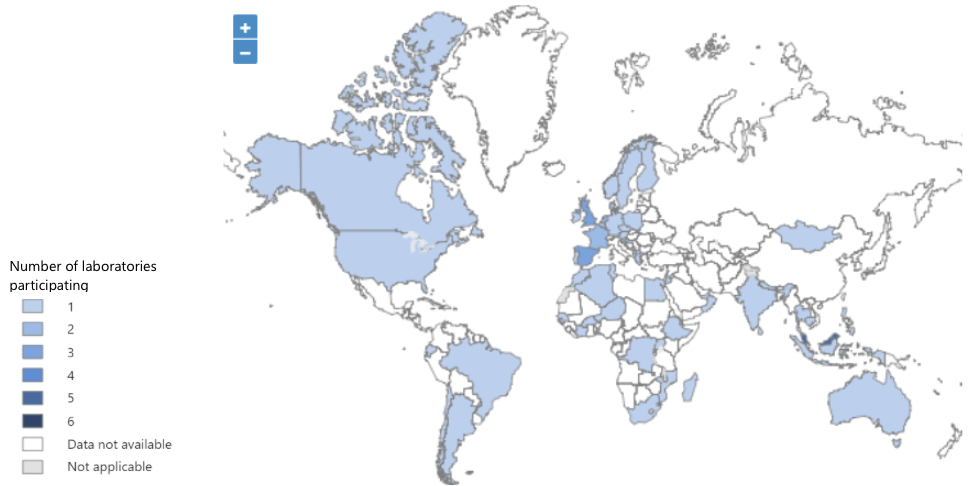

Supplement: Supplementary file 2 — Figure S1B: Countries submitting results for the fourth (2024) EQA. [file IRV-20-e70302-s001.png]

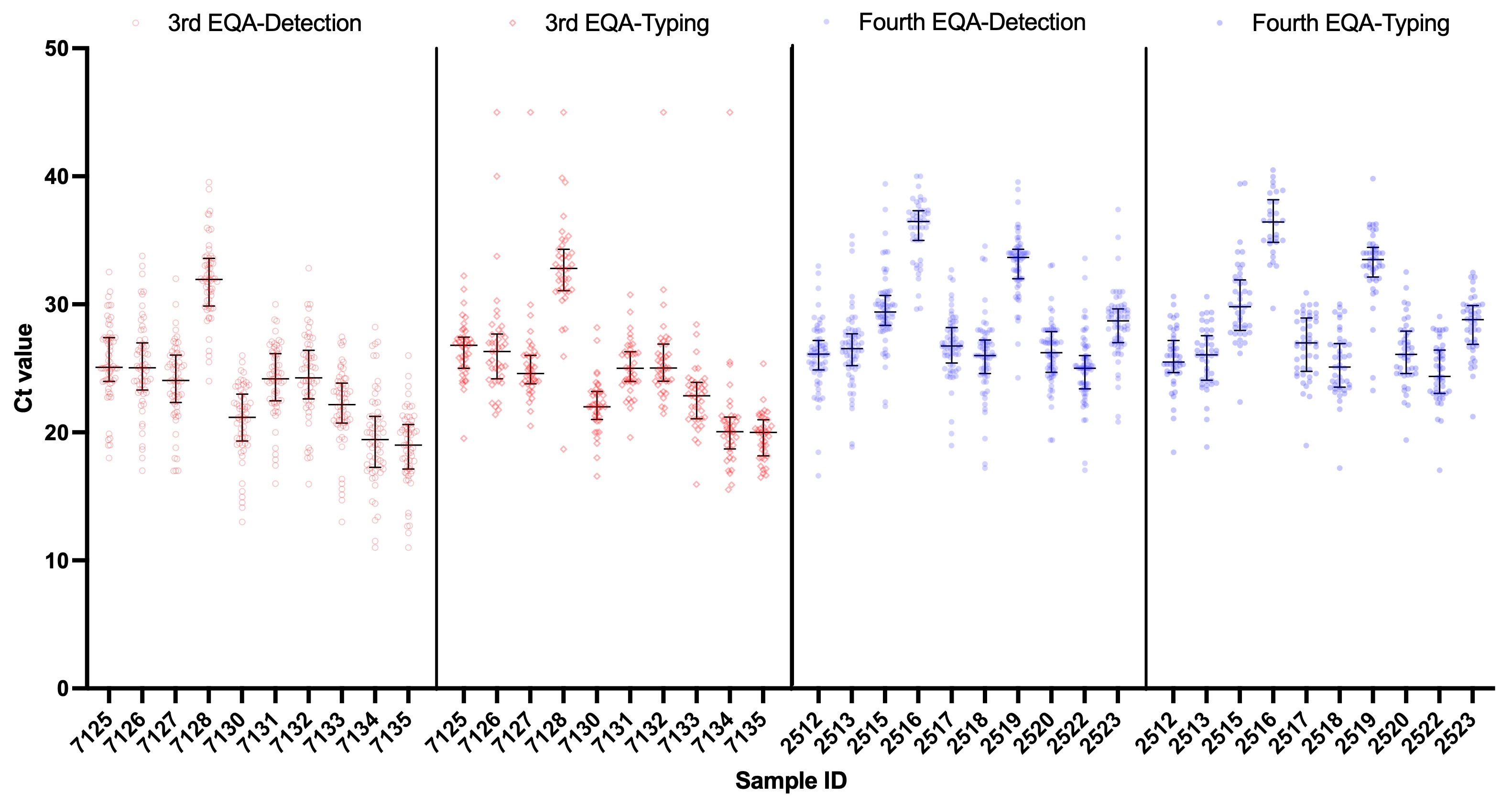

Supplement: Supplementary file 3 — Figure S2: Ct value distribution of all participants in the third and fourth EQAs. [file IRV-20-e70302-s002.jpeg]
