# Supplementary material for: Results From the Third and Fourth WHO External Quality Assessments for the Molecular Detection of Respiratory Syncytial Virus
Source: Influenza Other Respir Viruses. 2026 Aug 2;20(8):e70302. doi: 10.1111/irv.70302 (PMC13429278; doi:10.1111/irv.70302)
Supplement: Supplementary file 4 — Table S1: Sample intended results, Ct values, and detection scores for the third and fourth EQAs. [file IRV-20-e70302-s003.docx]

**Supplementary Table 1. Sample intended results, Ct values, and detection scores for the third and fourth EQAs**

1. **Third EQA**

| Sample ID | Intended results | Reference laboratory  Ct value | Mean reported  Ct-value by participants | Overall RSV detection score |
| --- | --- | --- | --- | --- |
| 7124 | Influenza A and SARS-CoV-2 | N/A | NA | 90% |
| 7125 | RSV A | 24.9* | 25.45 | 99% |
| 7126 | RSV B | 26.9 | 25.24 | 100% |
| 7127 | RSV B | 25 | 23.92 | 100% |
| 7128 | RSV B | 33.0* | 31.92 | 97% |
| 7129 | No virus | N/A | NA | 92% |
| 7130 | RSV A | 21.3 | 20.89 | 99% |
| 7131 | RSV A | 23.1 | 24.07 | 100% |
| 7132 | RSV B | 25.9 | 24.45 | 99% |
| 7133 | RSV A | 22.5 | 21.97 | 100% |
| 7134 | RSV B | 23.1 | 19.57 | 100% |
| 7135 | RSV A | 17.8 | 18.83 | 100% |

**B. Fourth EQA**

| Sample ID | Intended results | Reference laboratory  Ct value | Mean reported  Ct-value by participants | Overall RSV detection score |
| --- | --- | --- | --- | --- |
| 2512 | RSV A | 24.04 | 25.98 | 100% |
| 2513 | RSV B | 25.5 | 26.55 | 99% |
| 2514 | No virus | NA | NA | 93% |
| 2515 | RSV B | 27.7 | 29.62 | 100% |
| 2516 | RSV B | 28.4 | 35.79 | 73% |
| 2517 | RSV A | 24.6 | 26.78 | 100% |
| 2518 | RSV B | 23.7 | 25.95 | 100% |
| 2519 | RSV A | 30.9 | 33.18 | 99% |
| 2520 | RSV A | 24.1 | 26.21 | 100% |
| 2521 | Influenza A and SARS-CoV-2 | NA | NA | 92% |
| 2522 | RSV A | 22.9 | 24.97 | 100% |
| 2523 | RSV B | 28.1 | 28.43 | 100% |
